# Chewing and Drinking Activity during Transition Period and Lactation in Dairy Cows Fed Partial Mixed Rations

**DOI:** 10.3390/ani9121088

**Published:** 2019-12-05

**Authors:** Viktoria Brandstetter, Viktoria Neubauer, Elke Humer, Iris Kröger, Qendrim Zebeli

**Affiliations:** 1 Institute of Animal Nutrition and Functional Plant Compounds, University of Veterinary Medicine Vienna, 1210 Vienna, Austria; brandstetter.viktoria@gmail.com (V.B.); viktoria.neubauer@vetmeduni.ac.at (V.N.); elke.humer@vetmeduni.ac.at (E.H.); iris_kroeger@gmx.de (I.K.); 2 Institute for Food Safety, Food Technology and Veterinary Public Health—Unit for Food Microbiology, University of Veterinary Medicine Vienna, 1210 Vienna, Austria; 3 FFoQSI GmbH—Austrian Competence Centre for Feed and Food Quality, Safety & Innovation, 3430 Tulln, Austria

**Keywords:** simmental, dairy cattle, chewing activity, rumination-halters, calving

## Abstract

**Simple Summary:**

It is common to feed cows varying levels of forage fibre in the time span before calving to lactation. The resulting changes in chewing time may help to evaluate if diets have adequate fibre content. Using rumination-halters to measure the chewing activities in dairy cows, we found diminished rumination and eating activity, especially around parturition. This indicates ruminal buffering insufficiency and a greater risk for rumen acidification during this period. In addition, reduced eating time in early-lactation cows was accompanied by reduced drinking time. We suggest that monitoring of chewing activity can be useful to assess rumen disorder risks of the cows during the transition period and rumination-halters may also be used as a tool to identify cows which are about to calve.

**Abstract:**

Dairy cows need sufficient physically effective fibre (peNDF) in their diet to induce chewing with the latter stimulating salivation and maintaining rumen health. Thus, monitoring of chewing activity can be a non-invasive tool to assess fibre adequacy, and thus helping in the optimization of the diet. The objective of this study was to investigate and compare chewing activities of cows during transition period and in the course of lactation. Simmental dairy cows, in four different production groups such as dry period (from 8 to 6 weeks ante-calving), calving (24 h before and after calving), early-lactation (7–60 days in milk), and mid-lactation (60–120 days in milk) were used in the study. Cows were fed partial mixed rations supplemented with different amounts of concentrates. The chewing and drinking activity were recorded using rumination-halters (RumiWatch System, Itin+Hoch GmbH, Liestal, Switzerland). Feed data analysis showed that the peNDF content of the partial mixed ration (PMR) was highest during dry period, decreased around parturition, reaching the nadir in the lactation, in all cases, however, exceeding the peNDF requirements. Chewing data analysis showed that rumination time decreased (*p* < 0.05) in the time around parturition (from 460 min/d during dry period to 363 min/d 24 h before calving) and increased again in early-lactation (505 min/d), reaching a maximum in mid-lactation (515 min/d). Eating time was lowest for cows during early-lactation (342 min/d) and the highest for those in mid-lactation (462 min/d). Moreover, early-lactation cows spent less time (*p* < 0.05) drinking (8 min/d) compared to other groups (e.g., 24 min/d the day before calving and 20 min/d postpartum). Monitoring of chewing activity might be a useful tool to assess rumen disorder risks and welfare of the cows during the transition period. It further shows promising results to be used as a tool to identify cows that are shortly before calving.

## 1. Introduction

Chewing is an essential physiological process in cattle. Cattle need long and intensive chewing periods that promote saliva secretion and reduction of feed particle size, thus, stimulating nutrient degradation while maintaining rumen health [1]. Indeed, the length of the chewing periods reflects secretion of alkaline saliva and rumen buffering, being a good indicator of rumen health status [2]. Chewing consists of eating and rumination activity and both are strongly dependent on the diet, especially on the content of physically effective fibre (peNDF) and concentrate type fed [1]. Dairy cows have high energy demands, and, therefore, rations rich in readily degradable concentrates are typically fed to them, but at the same time these rations are low in peNDF. It is a challenge for dairy nutritionists to find an optimal balance between peNDF and readily degradable carbohydrates in the diet to ensure high milk production and at the same time prevent rumen metabolic disorders [1]. The diets low in peNDF provide less stimuli for chewing [1], and therefore may result in decreased chewing activity. Failure of an optimal balance between peNDF and readily degradable carbohydrates in the diet often leads to subacute ruminal acidosis (SARA), a prevalent metabolic disorder of high-producing dairy herds. Referring to Plaizier et al. [3], consequences of SARA may also include feed intake depression, reduced fibre digestion, milk fat depression, diarrhoea, laminitis, liver abscesses, and other secondary diseases. Thus, monitoring the variation in chewing activity can be helpful to evaluate structural fibre adequateness in the diets and adapt the diet in a way to prevent rumen metabolic disorders [4,5]. Because chewing is also affected by other factors, e.g., feed availability, cow health, and cow comfort [6,7,8], monitoring of chewing activity might help in identifying such disorders in an early phase. Because of an increase in free-stall barns and increased numbers of cows per farm, the supervision of eating and ruminating behaviour of dairy cows has become more difficult, and visual observations are time-consuming. Thus, the use of digital technologies like sensors to monitor the chewing activity of dairy cows can support management decisions and provide information about animal behaviour and health.

When cows are fed varying levels of peNDF during transition and in the course of lactation, their chewing activity is expected to differ. Recent studies, which examined chewing behaviour around parturition, mostly fed total mixed rations (TMR) of different forage:concentrate (F:C) ratios, and hence different peNDF contents. Because TMR contain all ingredients (i.e., forages and concentrates) at the same time, it is easier to measure their peNDF content and assess their responses in cows. Indeed, requirements for peNDF have been defined for cows fed TMR [1]. In contrast to TMR feeding, the partial mixed ration (PMR) feeding system basically consists in feeding only a minor part concentrates with forages, enough that PMR meets energy and nutrient requirements for maintenance plus a limited milk production level (i.e., 15–20 kg milk/d). Individual cows are then supplemented with additional amounts of concentrates based on the daily milk production, which is a much higher amount than the concentrate included in the PMR. This feeding system is appropriate for small- and medium-scale dairy farms (up to 70–80 cows), such as in Austria, and in farms using automatic milking robots. Therefore estimating fibre adequacy in PMR feeding and meeting the peNDF recommendations for cows is more challenging. Also, it is lesser known how PMR feeding affects chewing behaviour around parturition and in the course of lactation. To fill this gap, the primary purpose of our study is to investigate and compare the chewing activities during the transition period and different lactation groups in cows fed PMR with additional concentrates under field conditions. We hypothesised that chewing activity reflects changes of peNDF content in the consumed diet, being highest during dry cow PMR feeding period and lowest during early lactation PMR feeding, and this will indicate a deficiency in peNDF supply for these cows. Moreover, we evaluated the time spent drinking in parallel with the chewing dynamics in these cows. Water intake plays a key role in animal health, behaviour, milk production, control of body temperature, and metabolic functions in dairy cows [9].

## 2. Materials and Methods

### 2.1. Animals and Experimental Design

This trial was conducted on a typical dairy farm in Lower Austria with 33 Simmental dairy cows. The experiment was a randomized block design with cows in four different production groups (*n* = 5/group) including pregnant dry cows (from eight to six weeks before calving), cows around parturition (24 h antepartum and 24 h postpartum), as well as cows during early- (7 till 60 days in milk) and mid-lactation (60 till 120 days in milk). Cows of the same group were distributed along four consecutive recording blocks (*n* = 5), each lasting for two full days in order to avoid a confounding effect by time.

Dry cows were housed in a tie-stall with straw bedding, while lactating cows were kept together in a free-stall barn with straw beddings. Because the farm did not a special maternity pen, cows were kept to calved in their dry-cow stalls. About two days after calving, the cows were moved to the free-stall barn. All animals were permanently housed inside without access to pasture.

All procedures involving animal handling and treatment were conducted according to the approval of the institutional ethics committee of the University of Veterinary Medicine (Vetmeduni) Vienna and the national authority of Austria according to §26 of the Law for Animal Experiments, Tierversuchsgesetz 2012-TVG (ETK-13/07/2017).

### 2.2. Feeding

Dry cows were fed a PMR containing grass-legume silage and hay, supplemented with 1000 g concentrate for dry cows per animal each day (Table 1). Two weeks before the expected calving date, concentrate was continuously and individually increased to 3 kg/d until the cows were moved to the free-stall barn. Therefore, cows of the group 24 h before and after calving were fed the dry PMR supplemented with approximately 3 kg of concentrate (Concentrate for dry cows, concentrate for lactating cows, and grain mix in an equal ratio) per cow per day. Once the cows were moved to the free-stall barn, they were switched to the lactation feeding regimen. Early- and mid-lactation cows were also fed a PMR containing the same grass-legume silage and hay, supplemented with a commercial concentrate mixture for lactating cows and an on-farm made grain mix as an energy supplement (Table 1) based on the daily milk yield. The difference in the amounts of supplemented concentrate was insignificant between early- and mid-lactation cows (5.2 vs. 5.0 kg/d). The PMR was offered ad libitum to the cows. Components of the PMR were blended short before feeding it, using a feed mixing truck (Model BvL V-Mix 5-1S Agilo). Fresh PMR and the supplemented concentrate were offered twice daily in the morning and afternoon, with an average of 3.9 kg (early-lactation) and 3.8 kg (mid-lactation) concentrated feed per cow and meal. All cows had continuously free access to water throughout the trial. In the tie-stalls automatic drinking troughs for individual cows were available, whereas in the free-stall barn two large water troughs (2 × 1 × 1 m) for all cows were placed at the opposite side of the feeding table.

### 2.3. Feed Sampling and Analyses

Samples of the PMR and ingredients were taken once per test group during each recording block for chemical analysis of dry matter (DM), ash, crude protein (CP), ether extracts (EE), neutral detergent fibre (NDF) and acid detergent fibre (ADF). Analyses were conducted at the laboratory of *the* Institute of Animal Nutrition and Functional Plant Compounds, following the guidelines of the VDLUFA (2012). We also used the Penn State Forage Particle Separator (PSPS) [11], consisting of three sieves (aperture size of 19.0, 8.0, and 1.18 mm) and the bottom pan (<1.18 mm particles) to quantitatively determine the particle size of the forages and the PMR. This was always performed by the same person in the same manner. The supplemented concentrate was pelleted, and particle size was assumed to be fine (<8.0 mm). As recommended by Humer et al. [10], for each kilogram of additional concentrate to a PMR, 2% of the fraction >19.0 mm have to be subtracted and added to the fraction of particles <8.0 mm. In our case (approximately 5 kg of concentrate per cow each day; Table 1), the fraction >19.0 mm decreases by 10%, whereas the fraction of particles <8.0 mm increases by 10%. The physically effective neutral detergent fibre (peNDF) values were calculated as described by Kononoff et al. [11] and are shown in Table 1.

### 2.4. Measurement of Chewing and Water Intake Activity

During determination of chewing and water intake activity, each cow of the recording block was fitted with a RumiWatch halter, containing a noseband sensor as functional unit (Swiss Patent CH 700 494 B1, Agroscope, Ettenhausen, Switzerland; manufactured and distributed by Itin+Hoch GmbH, Liestal, Switzerland). Kröger et al. [12] reported the functioning, data recording, and accuracy of measurements by the RumiWatch halters in detail. The chewing activity was recorded during two consecutive days (exactly 48 h from the moment of accurate fit and confirmation of data transfer), to enable repeated observations for each animal. The measurements of all recording blocks were done within a period of 12 consecutive days (including days of changing between recording blocks). Summaries of the recorded data were read out wireless daily, using an USB antenna connected to the laptop as receiving device. This was done without interrupting the measurement, to control functionality and fit. After each measurement (two full days), raw data files were transferred manually from the SD card to a computer. RumiWatch halters were used to detect diurnal ruminating and eating patterns (min/d), duration of eating (min/d), ruminating (min/d), total chewing time (min/d), the number of ruminating chews (n/min), eating chews (n/min) and ruminating boli (n/d), ruminating chews per bolus (n/bolus), furthermore drinking time (min/d) and drinking gulps (n/d). The recorded data was evaluated with the RumiWatch Manager (Version 2.2.0.0). Using the corresponding data converter (RWC Version V0.7.4.5), daily data was converted into one-hour classification summaries for each animal-specific data file. The data as a sum of 24 h was used for further interpretations. The noseband sensor was well tolerated by all cows and no skin irritations occurred during our feeding experiment.

### 2.5. Milk Production Data

Cows were milked in the milking parlour twice daily at 05:00 and 17:00 h. For milk component analysis and milk yield, milk samples were collected on three consecutive milking-times (morning and evening milking) of early- and mid-lactation cows at the same days as their chewing activity measurements took place. Because of the diurnal variations of milk components between morning and evening milking, we used mean values of the three measurements per cow to provide a more accurate value. Milk components including milk fat, milk protein, lactose, fat-free DM, milk urea nitrogen (MUN), pH, somatic cell count (SCC) and fat:protein ratio were determined in the milk laboratory of lower Austria (LKV, Zwettl, Austria). Milk yield was measured on site using TRU-Test-Milkmeters, provided by the LKV.

### 2.6. Statistical Analyses

Statistical analysis of chewing activity was carried out using the MIXED procedure of SAS (Version 9.4, SAS Institute Inc., Cary, NC, USA). The group was considered as fixed effect in the ANOVA, and measurements collected at different days on the same cow within a group were considered as repeated measures in the model with a variance components (VC) variance-covariance matrix. Animals were considered as random effects and cow was the experimental unit. The ANOVA of milk component data was also performed using the mixed procedure of SAS (Version 9.4, SAS Institute Inc., Cary, NC, USA). For each variable tested, group and recording block were considered as fixed effects and animal was considered as random effect. The approximation of degrees of freedom was performed according to Kenward-Roger. Least square means were computed and multiple differences among the means were performed using the pdiff option of the SAS. Comparisons among means were declared significant at *p* ≤ 0.05, a tendency for differences among means was considered at *p* ≤ 0.10.

## 3. Results

### 3.1. Feed and Water Intake and Physical Structure of the Diet

Particle size distribution indicated a larger portion of particles >19.0 mm in the PMR (72%), compared to the reference values [13], resulting in 27.5% peNDF > 8.0 mm (Table 1). Only a small proportion of the very fine particles (<1.18 mm) was measured in the PMR (4%). However, because of the adaption for each kilogram of additional concentrate to a PMR, the fraction >19.0 mm decreases to 62%, whereas the fraction of particles <8.0 mm increases to 9%, which lowers the absolute content of the large particles in the consumed diet during the lactation period. Also, both the grass-legume silage and hay contained larger proportions of the fraction >19.0 mm (87 and 90%, respectively) than recommended [14].

**Figure 1:**
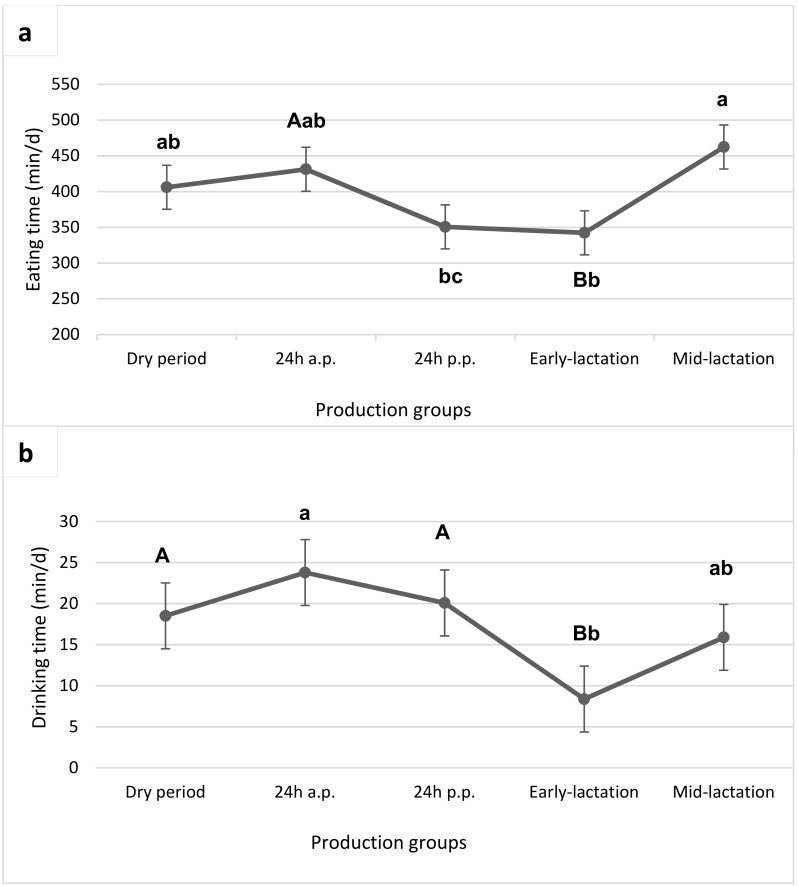
Eating time (**a**) and drinking time (**b**) of cows in different groups (Dry period, 24 h antepartum, 24 h postpartum, early-lactation, mid-lactation). Groups bearing different lowercase letters differ at *p* < 0.05 and capitals differ at *p* < 0.10.

Figure 1a shows the time spent eating from cows of different groups. Eating time during the dry period (406 min/d) did not significantly differ from the 24 h antepartum (431 min/d). It was lowest postpartum (351 min/d) and during early-lactation (342 min/d), and highest during mid-lactation (462 min/d). Early-lactation cows spent significantly less time eating than mid-lactation cows did (342 vs. 462 min/d, respectively) and tended to spend less time eating than the cows 24 h before their parturition. The time spent eating of the cows during 24 h postpartum did not differ from cows antepartum or cows during early-lactation, but postpartum cows spent less time eating than those in mid-lactation (351 vs. 462 min/d). Further, cows around parturition spent more time drinking (24 min/d antepartum, 20 min/d postpartum), than cows during early-lactation (8 min/d; Figure 1b). Also drinking gulps were lowest in early-lactation cows (132 vs. 240 gulps/d for mid-lactation cows).

### 3.2. Chewing and Rumination Data

**Figure 2:**
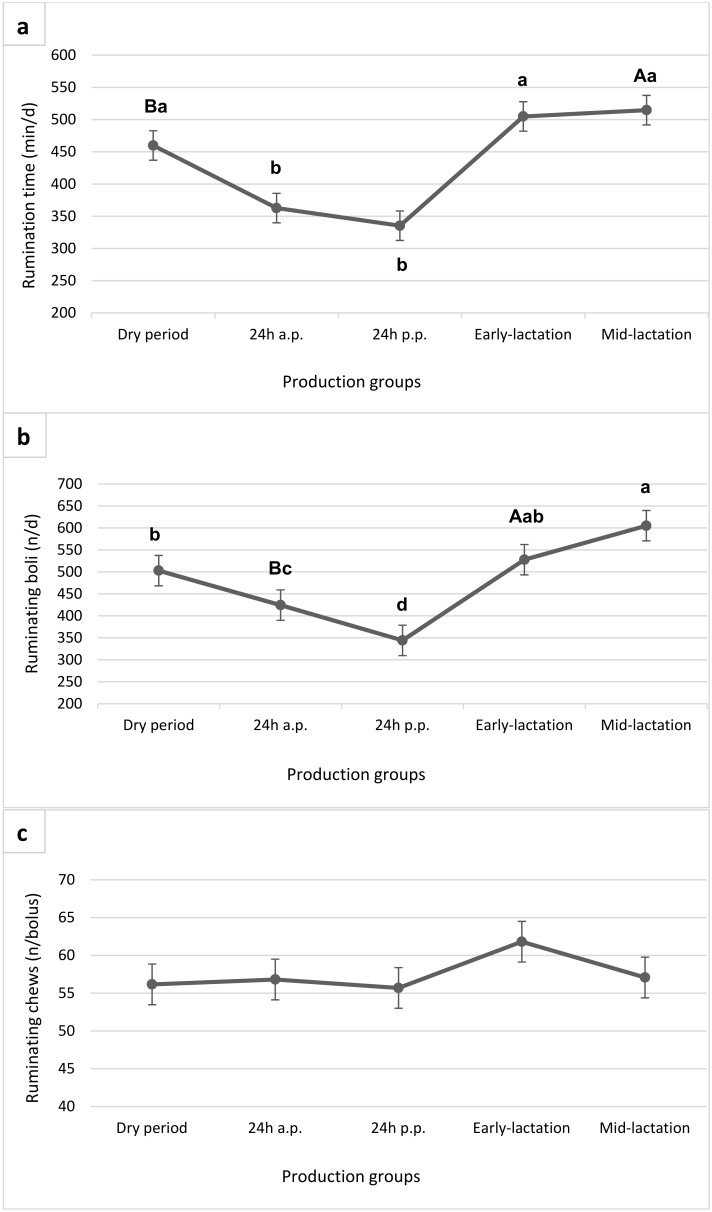
Rumination time (**a**) ruminating boli (**b**) and ruminating chews (**c**) of cows in different production groups. Least square means (LSM) bearing different lowercase letters differ at *p* < 0.05 and capitals differ at *p* < 0.10.

Rumination time (a) and ruminating boli (b) during the dry period were 460 min/d and 503 boli/d, whereas cows 24 h before calving ruminated 363 min/d and had 424 boli/d and cows postpartum ruminated 335 min/d and had 344 boli/d, respectively. During early-lactation the rumination time was 505 min/d and the number of ruminating boli 528 per day, however we investigated the maximum values during the mid-lactation (515 min/d, 605 boli/d). The number of ruminating chews per bolus (Figure 2c), as well as per unit of time (min) did not differ among groups in our trial.

**Figure 3:**
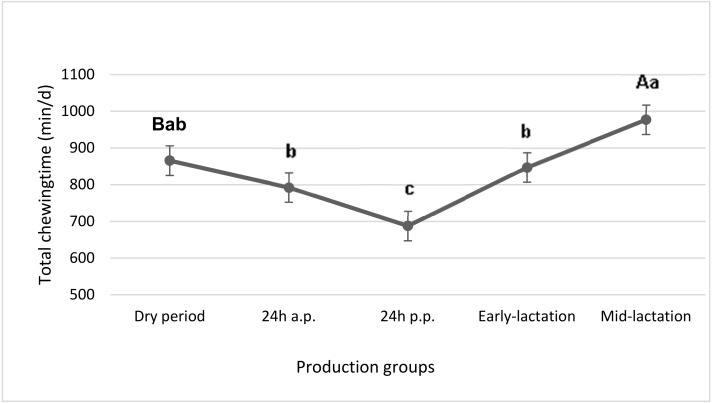
Total chewing time of cows in different groups. LSM bearing different lowercase letters differ at *p* < 0.05 and capitals differ at *p* < 0.10.

Total chewing time data, which is the sum of eating and rumination time, is shown in Figure 3. Data showed that it was lowest the day after calving (687.6 min/d), as against mid-lactation, where total time spent chewing was 977 min/d.

**Figure 4:**
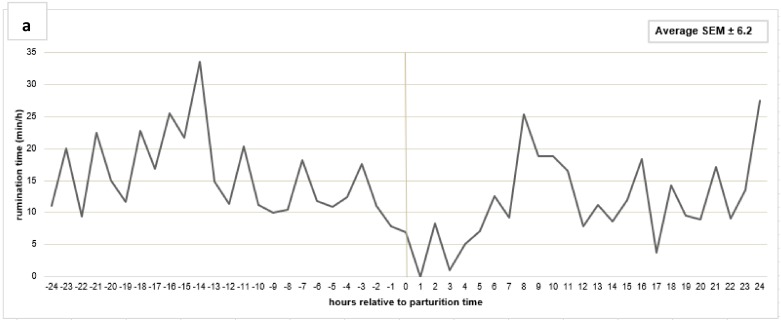

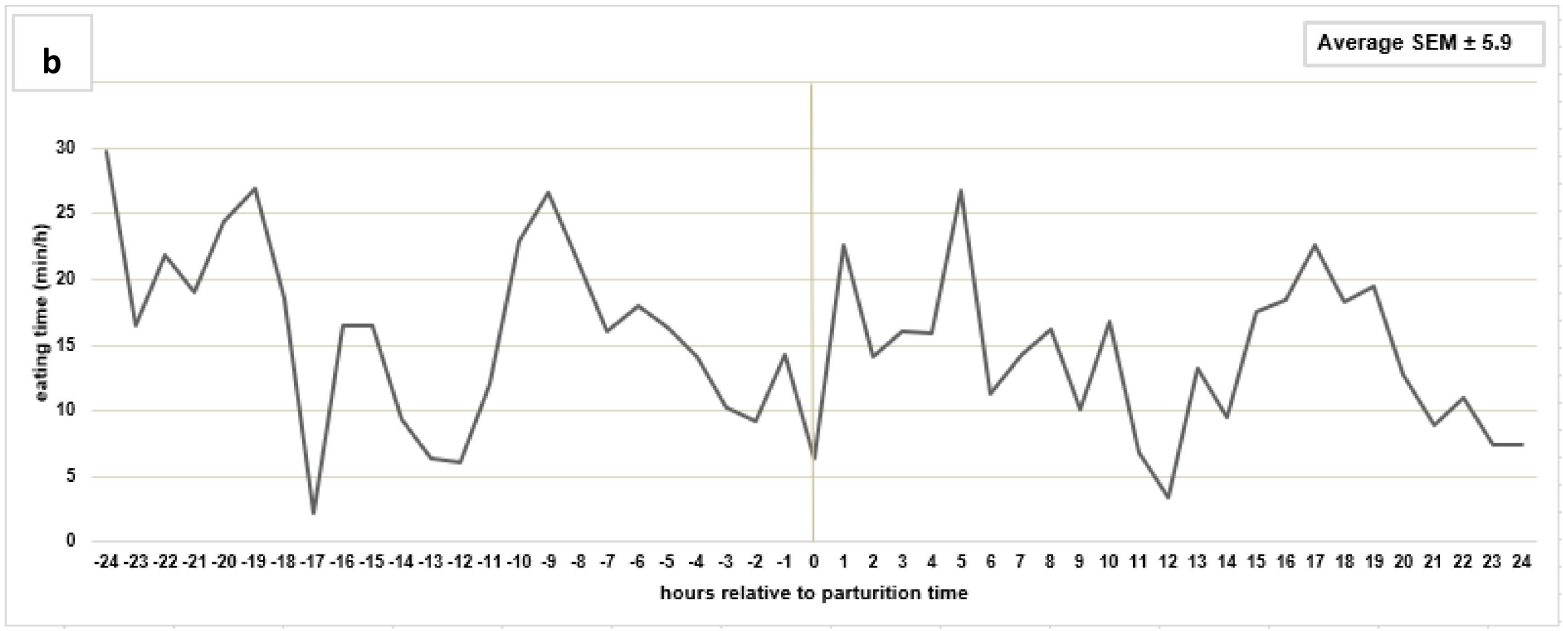
Changes in time spent ruminating (**a**) and eating (**b**) in one-hour-intervals from 24 h before (−24) to 24 h after calving (24). SEM standard error of mean.

For detail information about diurnal eating and rumination patterns around parturition, mean values per hour were calculated (Figure 4a,b). The time spent ruminating was lower 24 h before calving in comparison to the dry period (Figure 2a). In the hourly analysis 24 h before and after calving, there was a decreased rumination time 2 h before calving, and especially in the 5 h after the calving event. The rumination activity increased after 5 h postpartum back to the level antepartum but did not reach the time of dry cows (Figure 2a and Figure 4a). However, there was no clear drop in eating time pattern around parturition (Figure 4b).

### 3.3. Milk Production and Composition

Milk production of cows changed from 30.4 kg/d during early- to 27.9 kg/d during mid-lactation, indicating that cows reached their peak of lactation at <60 days postpartum. While milk fat was the same for early- and mid-lactation cows (on average 3.7%), protein tended to be higher (2.9 vs. 3.3%; *p* = 0.09) in mid-lactation cows (Table 2). Percentage of lactose in milk was significantly higher in early-lactation (*p* = 0.01). However, the percentage of fat-free DM, milk urea nitrogen (MUN), pH-value, somatic cell count (SCC), and fat:protein ratio were similar for both production groups (Table 2).

## 4. Discussion

This study aimed to investigate and compare the chewing activities of cows during the transition period and in the course of lactation under practical conditions.

### 4.1. Feed and Water Intake and Physical Structure of the Diet

Analysis of the feeding phases did not show a significant decrease in the mean eating time 24 h antepartum compared to the dry period, however a numerical decrease of 80 min/d eating time from 24 h before to after calving. The shortest time spent feeding was 24 h postpartum and during early-lactation (351 min/d postpartum, 342 min/d in early-lactation). It was previously reported that the time spent feeding is higher in the ante-calving period, compared to the post-calving period; however literature showed the results in Holstein dairy cows [15,16,17]. Early-lactation cows spent significantly less time eating than mid-lactation cows (342 vs. 462 min/d). This finding can be explained by higher total DMI in mid-lactation. This has also been reported by DeVries et al. [18], who showed longer eating times with 94 DIM, compared to 35 DIM.

In this trial, cows were slowly shifted to an energy-denser diet from about two weeks before calving by increasing the concentrates in the ration. Increasing the concentrate usually results in faster and higher DMI. Although the DMI was not determined in the current study, we speculate that the lower eating times in the time post-calving are due to faster consumption of energy denser, low-forage diets compared to the ante-calving period [19,20]. Increasing the amount of concentrate is important to increase the energy intake; however, increasing the concentrate amounts may occur at the expense of the forage-rich PMR intake, which is expected to lower the overall intake of the peNDF [1]. However, the PMR in our trial was of minor acidotic risk because of high peNDF content. Interestingly, despite shorter eating times, the early-lactation cows in this trial consumed the same amount of concentrates as mid-lactation cows, suggesting that the ratio of concentrate to forage in early-lactation cows was higher, and thus the PMR intake was less than in mid-lactation cows. On the other hand, early lactation cows showed the same rumination time and the same number of ruminating boli as mid-lactation cows. Therefore, it is possible that the intake of peNDF exceeded the threshold to affect the rumination activity both in early lactation and mid-lactation cows [21].

The hourly eating time did not show clear evidence for a decrease around parturition, which would be an indicator for the calving event. In contrast to our findings, Schirmann et al. [17] and Miedema et al. [22] found decreased eating times eight and six hours before calving, which remained suppressed in the two days after calving. Differences among studies can be explained by different housing (moving cows to maternity pens) or feeding strategies.

Overall, calving itself seems to have a greater impact on the rumination than on eating time as there is a distinct drop in rumination time 24 h ante as well as post-calving compared to the dry period, whereas the decrease in eating time mainly occurs postpartum. We ascertained that also the number of eating chews was relatively stable antepartum but declined postpartum, which might be a negative impact of the increasing amount of concentrates around calving and the event of calving itself, harbouring possible discomfort for the cow.

The longer drinking times around calving (24 min/d antepartum, 20 min/d postpartum) in comparison to the other periods, are a sign that cows are thirstier because of exhaustion, stress, and water loss during calving and colostrum production. Huzzey et al. [16] observed 5.5 ± 0.29 min/d ante- and 6.8 ± 0.29 min/d post-calving. In their study, total drinking time increased, when cows moved from ante- to the post-calving period. This is also consistent with other literature since big water losses are happening due to high milk production postpartum, especially in early-lactation [23,24]. Therefore, we would have expected an increase in drinking time postpartum with maximum minutes per day in early-lactation, instead of a reduction as shown in our observations (8 min/d during early-lactation). However, shorter drinking time might have more water ingested if the gulps are more frequent or larger, so results of drinking time must be considered critically. Moreover, water consumption is highly affected by many factors, such as water loss due to milk production, ambient temperature changes, feed intake, dry matter of the diets, consumption of sodium and potassium, as well as physiological factors and diseases. The high milk production in early-lactation cows should have rather increased drinking time during early-lactation. We can also exclude temperature as an influencer to drinking behaviour here, because our trial was conducted at a short period of time, and each cow was exposed to the same conditions (15–25 °C, August). Sodium-intake cannot explain a decrease in drinking time postpartum either, because it is included in the mixed ration as mineral-vitamin-premix for lactating cows (11.5% Na) and as a concentrate for dry cows (1.4% Na). There were no salt lick blocks available. Furthermore, there were no detrimental changes in the diets between early- and mid-lactation, or between 24 h antepartum and postpartum. Thus, dry matter, sodium and potassium content cannot explain the decreased drinking time postpartum and in early-lactation. What we did not take into account are measurable physiological parameters, as for example the hormone aldosterone is involved in the regulation of the electrolyte and water balance in organisms. It is secreted by the adrenal cortex and increases the renal retention of sodium and the excretion of potassium. It could indeed passively change drinking behaviour. Another aspect that has to be considered is that drinking time might be influenced by welfare, housing-conditions and watering systems, hence dry cows were housed in tie-stalls with automatic drinking troughs for individual cows, whereas in the free-stall barn two huge water troughs (2 × 1 × 1 m) for all cows were placed at the opposite side of the feeding table. Different watering systems might have an impact on drinking duration measurements. While automatic drinking troughs during dry period and around calving are relatively small, the huge water troughs of early- and mid-lactation cows might allow them to consume more water. Also, Pinheiro et al. [25] showed, that cows spent more time drinking, consumed more water and took more sips, when they had access to a higher, larger trough. However, this is not in line with the decreased drinking times of early-lactation cows in our trial. It is questionable, whether cows drinking from huge troughs can consume more water in a shorter time span than cows drinking from smaller automatic drinking troughs. This could explain shorter drinking times of early-lactation cows, without necessarily indicating less water consumption. However, further research on different watering systems is needed to ascertain this assumption.

Moving the cows from tie-stalls to the free-stall barn after calving could have influenced the drinking behaviour because of new grouping and hierarchical restructuring. However, since postpartum and during early-lactation, cows showed depressed feed intake and less eating time per day, we suggest that these factors influenced the drinking time the most. However, we cannot tell whether feed intake influenced drinking time or drinking time influenced feed intake, so this question needs further research to be answered. Moreover, previous studies [26] showed that the RumiWatch system is error-prone when measuring drinking behaviour in cows. However, this reflects more the challenge in measuring drinking in general than merely with the RumiWatch system. Further validation of the RumiWatch system and continuing development, especially for drinking measurements is needed.

The grass-legume silage fed to the dry cows contained more large particles in the upper sieve (87.0%), whereas, in the middle and lower sieves there was less content than designated (6.2 and 6.1%). Referring to the data of Heinrichs and Kononoff [14] for grass silage, 10 to 20% of the crop should be >19.0 mm (upper sieve), 45 to 75% should be in the 8-0-19.0-mm sieve (middle sieve), 20 to 40% in the 1.18-8.0-mm sieve (lower sieve) and <5% should be <1.18 mm (bottom pan). The outcomes for the PMR were similar, as Humer et al. [13] recommended 15 to 25% of the particles in the upper, 35 to 65% in the middle, 15 to 25% in the lower sieve, and <8% in the bottom pan. A too high proportion of particles >19.0 mm should be avoided to prevent sorting, decreased DMI, and decreased absolute intake of peNDF. The results of the peNDF confirm that cows were well supplied with structure, because values should be between 14 and 18% [1]. To sum up, the ration is too rich in long particles. This can lead to depressed eating time, decreased DMI, lower passage rate and feed efficiency, digestibility, and nutrient uptake [1,19,27,28,29,30,31]. Also sorting behaviour can be more distinct, as the amount of material retained on the 19.0-mm sieve of the PSPS correlates to sorting behaviour [19,32]. Because of the results of Kononoff et al. [33] a moderate particle-size reduction in our diet could indeed decrease sorting behaviour and concurrently increase DMI by 1 to 2 kg/d. In their trial, they used long chopped forage (theoretical length of cut = 22.3 mm; 31.4% >19.0 mm) and short chopped forage (theoretical length of cut = 4.8 mm; 3% >19.0 mm) and DMI (kg/d) increased from 20.1 to 23.4 kg/d.

### 4.2. Chewing and Rumination Data

In general, data of chewing behaviour revealed strong variation during transition period and in the course of lactation. Beauchemin et al. [34] ascertained an average time of 426 ± 16 min/d for rumination time of cows fed a PMR. During dry period and lactation, values were above this benchmark; however, the day before and especially after calving, ruminating activity decreased below it. Additionally to rumination time, also ruminating boli decreased in the 24 h antepartum, corresponding the data of Fadul et al. [35]. The transition period is a critical time in the lactation cycle of dairy cows as they experience changes in nutritional needs, physiological processes (e.g., hormones) and social conditions, and are more vulnerable to infections and metabolic disorders [36] Also, Schirmann et al. [17] reported a significant decline in rumination time 24 h before calving, and even stronger in the 24-hour period following the expulsion of the calf. In their study, the decrease in rumination time was accompanied by decreased DMI and feeding time, corresponding to our data. Another illness, which usually occurs postpartum and during early-lactation, and may decrease chewing activity, is ketosis (clinical/subclinical). Kaufman et al. [37] found 25 ± 12.8 min/d less rumination in multiparous cows with ketosis (without other health problems). Schirmann et al. [17] also depict that regrouping and diet changes (especially NDF) before or after calving can have an impact on the rumination behaviour. Cows in our study were not moved to special maternity pens before calving but moved back to the free-stall barn a few days after calving. Diet changed after moving to the free-stall barn, when the cows received the ration for lactation cows. Thus, these two factors could not have an impact on depressed rumination behaviour the day before or the 24 h after calving. We also considered diurnal rumination patterns around parturition to receive detailed information about when exactly the reduction of rumination time occurs. Schirmann et al. [17] were the first to describe patterns of rumination and feeding behaviour around parturition, taking the time when the calf is born into account. According to their findings, our data also suggests that time spent ruminating decreases within 24 h before calving, being distinct especially two to three h before the expulsion of the calf and reaching a minimum in the five hours after calving. Rumination time increased after six to eight hours following parturition but did not return to baseline values until 24 h after calving, which also matches the results of Schirmann et al. [17]. These findings suggest that monitoring of rumination behaviour could be a useful tool to predict calving times under farm conditions. Also, Fadul et al. [35] depicted that the RumiWatch system can predict the calving event within the next 3 h with a high level of accuracy, especially when combining the RumiWatch system noseband-sensor and the 3D-accelerometer. Caution is required in the use of daily rumination summaries, since one-hour classification summaries deliver more detailed information. Further research is needed to determine the reliable thresholds for decreased rumination time before calving, as calving-alarms could be useful on-farm tools.

Literature shows that with increasing F:C ratio, the particle size also increases, which leads to increased rumination time [19,38]. Interestingly in our study, rumination time during the dry period did not statistically differ from early-lactation, although relative fibre content of the dry period ration was higher than in early-lactation. It even tended to be lower during the dry period than in mid-lactation, which could be explained by less total feed intake of dry cows. However, Allen [2] suggested that above a threshold of 10 mm mean particle size, moderate or no further increase in rumination time is shown anymore. In addition, Zebeli et al. [5] showed physiological limits, beyond which the peNDF > 8.0 is not able to improve rumination and rumen buffering in lactating dairy cattle (peNDF > 8.0 up to 16.4–20.6%). Therefore, we cannot expect an increase in rumination time in our dry cows, hence already the early-lactation cows are oversupplied with structure. However, although rumination time during the dry period did not exceed early- or mid-lactation values, it was still within the range suggested by Beauchemin et al. [34]. As reported for rumination time, also ruminating chews (n/d) tended to be even lower during the dry period, compared to mid-lactation. The number of ruminating chews per unit of time (min) and bolus did not differ between groups in our trial. These parameters seem to be stable and not easily affected by the diet changes or calving, although it has been reported that low fibre content, small particle size and rising concentrate levels decrease ruminating chews per bolus and ruminating boli per day [4]. However, since the cows in our experiment were always well supplied with structure and the proportion of concentrate was moderate, we did not expect a significant decline of the ruminating chews per minute or bolus.

Beauchemin et al. [39] showed that total chewing time increased as dietary fibre concentration increased, mainly driven by increased rumination time. Also Fernandez et al. [40] suggested that the longer the particles, the higher the peNDF and the longer the time spent chewing. In our study, total chewing time did not differ between dry period and early-lactation. A statistical tendency is shown between dry period and mid-lactation. The decline in total chewing time from 24 h before calving to the day after calving was caused by the strong decrease in eating and rumination time.

In conclusion, especially rumination time decreased clearly in the hours around calving, thus monitoring of ruminating behaviour shows promise as a tool to identify cows that are before calving. This trial clearly shows that depressing effects of parturition on chewing activity become obvious and may be indicated by automatic monitoring. However, we have to consider that 24-hour-summaries have to be viewed with caution. Based on the trend towards high performing dairy farms nowadays, where cows are fed rations rich in concentrated feedstuff and often too little structure, decreases in e.g., rumination time would be even more distinct than in our trial, where cows were always well supplied with structure. Therefore ruminal buffering insufficiency during the time around parturition leads to a greater risk of rumen acidification in high-yielding dairy cows.

### 4.3. Milk Production and Composition

Mid-lactation cows had higher feed intake and lower milk yield, leading to a more sufficient energy supply, seen in higher milk protein values. In comparison, early-lactation cows had a milk protein of 2.9%, which is below the recommended threshold of 3.2, indicating dietary energy deficiency. However, the fat:protein ratio was 1.3 for early-lactation cows, being within the recommended physiological range of 1.1–1.5. Milk fat values were 3.7% for early-lactation, and 3.8% for mid-lactation cows, and therefore in an adequate range for Simmentals. These values are in line with the supply of peNDF that exceeded requirements in our study and therefore provide useful information about fibre supply and adequate diets for ruminants. However, especially milk fat values need to be interpreted with caution until around 90 DIM, because cows might be in an energy deficit status and mobilize body fat. Besides the interpretation of milk content values on herd level to receive feedback on the diet, it is also important to look at individual cow data to evaluate other risk factors. In one mid-lactation cow, which had obvious claw problems (lameness score 4/5; confirmed by the local vet), we found individual milk data with a milk protein of 2.6%, increased milk fat (4,4%) and an increased fat:protein ratio (1.7), indicating risk for ketosis because of insufficient energy intake and body fat mobilisation. This is supported by our data of chewing activities, where lowest eating time of this cow was 232 min/d and lowest rumination time 374 min/d, both undercutting the average values of the other mid-lactation cows in our trail. Our example shows, that lameness (pain), may result in increased recumbency, less food intake and therefore insufficient energy intake, as well as decreased chewing and drinking activity.

Taken together, monitoring of chewing activity is a useful tool to assess the rumen disorder risks and welfare of the cows during the transition period and lactation fed various PMR and supplemented with different amounts of concentrate. It further shows promising results of using chewing data as a tool to identify the cows that are about to calve. The chewing halters can replace time-consuming visual observations to record chewing and drinking behaviour in cattle. However, right now, the halters are still very expensive, and analysis of the data is complex, thus they are more likely for research purposes than for practical use on dairy farms today. For precision feeding reasons, further developments are needed especially in regards of coupling the data of chewing and drinking activity with diet and milk composition (fat and fat:protein ratio), which might be useful indicators of both fibre adequateness and energy level supply, as well as welfare and normal behaviour of dairy cows. In the future, it is also worth comparing the monitoring system used in the same experimental model but other breeds (e.g., Holstein, Jersey).

## Figures and Tables

**Table 1:** Ingredients and nutrient composition, and particle size distribution of partial mixed rations (PMR) and the supplemented concentrates fed to cows during transition period, around parturition, and during lactation.

	Dry Period	Parturition	Early-Lactation	Mid-Lactation
**Ingredients of PMR (% of DM, unless otherwise stated)**				
Grass-legume silage	90.4	90.4	84.6	84.6
Hay	9.6	9.6	5.7	5.7
Protein supplement ^1^	0	0	7.2	7.2
Grain mix ^2^	0	0	1.7	1.7
Mineral-vitamin premix ^3^	0	0	0.8	0.8
**Nutrient composition of the PMR** **(% of DM, unless otherwise stated)**				
DM (% of fresh feed)	43.5	43.5	40.7	40.7
Ash	10.5	10.5	10.4	10.4
Crude protein	16.7	16.7	17.7	17.7
Ether extracts	2.9	2.9	3.1	3.1
Neutral detergent fibre	40.4	40.4	33.2	33.2
Acid detergent fibre	22.9	22.9	19.2	19.2
**Particle size distribution (% of DM)**				
>19.0 mm	87.3	87.3	72.2	72.2
8.0–19.0 mm	6.0	6.0	10.7	10.7
1.18–8.0 mm	5.9	5.9	13.2	13.2
<1.18 mm	0.8	0.8	4.0	4.0
pef_8.0_^4^	93.3	93.3	82.9	82.9
peNDF_8.0_^5^	37.7	37.7	27.5	27.5
**Supplemented concentrate (g/cow/d)**				
Concentrate for dry cows ^6^	1000	1000	0	0
Concentrate for lactating cows ^7^	0	1000	2703	2603
Grain mix	0	1000	2530	2464
Total supplemented concentrate	1000	3000	5233	5067

^1^ RinderKombi 40 H (Garant Tiernahrung); contained: rapeseed meal, dried distillers grains and solubles (DDGS), sunflower meal, molasses, Ca-carbonate, Na-chloride, Mg-oxide; Composition: 7.5% ash, 40% crude protein, 4.5% ether extracts, 10% crude fibre, 7.05 MJ/kg NEL, 0.8% Ca, 0.95% P, 0.25% Na, 0.5% Mg; mineral-vitamin content (per kg): 10,000 IU of vitamin A, 2000 IU of vitamin D3, 20 mg of vitamin E, 70 mg of zinc, 40 mg of manganese, 15 mg of copper, 1.6 mg of iodine, 0.6 mg of cobalt, 0.4 mg of selenium. ^2^ Contained: wheat, barley, triticale, corn and consisted of (DM-basis): 88.0% dry matter, 2.4% ash, 11.3% crude protein, 2.4% ether extracts, 16.2% aNDF and 5.6% ADF. ^3^ Rinder Genuss plus (Ramikal); composition: 15% Ca, 4.5% P, 11.5% Na, 3.0% Mg, 1,000,000 IU of vitamin A/kg, 100,000 IU of vitamin D3/kg, 4000 mg of vitamin E/kg, 10,000 mg of zinc/kg, 6000 mg manganese/kg, 1000 mg copper/kg, 104 mg iodine/kg, 46 mg cobalt/kg, 50 mg selenium/kg, 2500 mg biotin/kg. ^4^ Physical effectiveness factor, determined as the proportion of particles retained on 19.0- and 8.0-mm sieves [10]. ^5^ The amount of particles retained on 19.0- and 8.0-mm sieves multiplied with the NDF of the feedstuff (in % of DM). ^6^ Vital-Kilo concentrate for dry cows (fixkraft); contained: wheat, wheat bran, linseed, barley, corn, Ca-carbonate, rapeseed meal, Mg-sulphate, molasses, Na-chloride; composition (estimated): 14.7% ash, 11% crude protein, 5.6% ether extracts, 4.5% crude fibre, 4.5% Ca, 4.5% P, 1.4% Na, 6000 IU of vitamin A/kg, 600 IU of vitamin D3/kg, 75 mg of vitamin E/kg, 94 mg copper E4/kg, 290 mg zinc E6/kg, 190 mg manganese/kg, 1.9 mg selenium/kg, 3.5 mg iodine/kg. ^7^ RinderKombi 25 (Garant Tiernahrung) contained: rapeseed meal, wheat bran, dried distillers grains and solubles (DDGS), corn gluten meal, molasses, wheat, Ca-carbonate, sunflower meal, Na-chloride, Mg-chloride; composition (estimated): 8.5% crude fibre, 6.7 MJ/kg NEL, 0.8% Ca, 0.95% P, 0.25% Na, 0.4% Mg; mineral-vitamin content (per kg): 10,000 IU of vitamin A, 2000 IU of vitamin D3, 20 mg of vitamin E, 70 mg of zinc, 40 mg of manganese, 15 mg of copper, 1.6 mg of iodine, 0.6 mg of cobalt, 0.4 mg of selenium.

**Table 2:** Milk yield and milk composition of cows in early- and mid-lactation.

Item	Early-Lactation	Mid-Lactation	SEM	*p*-Level
Milk yield (kg/d)	30.4	27.9	1.72	<0.01
Milk composition				
Fat (%)	3.7	3.8	0.25	0.82
Protein (%)	2.9	3.3	0.14	0.09
Lactose (%)	4.9	4.6	0.06	0.01
Fat-free DM (%)	8.50	8.65	0.13	0.44
Milk urea nitrogen (mg/L)	183	132	29.01	0.25
pH-value	6.63	6.56	0.03	0.13
Somatic cell count (SCC, cells/mL)	109,600	158,900	53,351	0.53
Fat:protein ratio	1.3	1.2	0.10	0.37
